# Dental Caries in Older Adults: A Narrative Review for Non-Dental Providers

**DOI:** 10.3390/ijerph23080965

**Published:** 2026-07-26

**Authors:** Martin S. Lipsky, Lucia Romero, Owen Cohen, Sajjan Dhasi, Lily Overman, Man Hung

**Affiliations:** 1 College of Dental Medicine, Roseman University of Health Sciences, South Jordan, UT 84095, USA; 2 Institute on Aging, Portland State University, Portland, OR 97201, USA; 3 OHSU-PSU School of Public Health, Portland State University, Portland, OR 97201, USA; 4 Division of Public Health, University of Utah, Salt Lake City, UT 84108, USA; 5 Eccles School of Business, University of Utah, Salt Lake City, UT 84112, USA

**Keywords:** dental caries, aged, xerostomia, prevention, oral health, non-dental providers

## Abstract

**Highlights:**

**Public health relevance—How does this work relate to a public health issue?**

- Dental caries is one of the most prevalent chronic diseases affecting older adults.
- Caries is an underrecognized contributor to pain, impaired nutrition, systemic illness, and reduced quality of life.

**Public health significance—Why is this work of significance to public health?**

- Non-dental providers encounter older adults regularly and have opportunities to identify risks long before advanced dental complications arise.

**Public health implications—What are the key implications or messages for practitioners, policy makers and/or researchers in public health?**

- Non-dental clinicians can reduce disease burden by reviewing medications, providing dietary and xerostomia counseling, recommending fluoride-based prevention, reinforcing good oral hygiene practices, and facilitating timely referral.

**Abstract:**

Dental caries is one of the most prevalent chronic diseases affecting older adults and remains an important, underrecognized contributor to pain, impaired nutrition, systemic illness, and reduced quality of life. Older adults access medical care more often than dental care, positioning non-dental clinicians to identify risk, initiate prevention, and facilitate timely referral. A narrative review of English-language literature was conducted using PubMed and Google Scholar. Search terms included “dental caries,” “root caries,” and “older adults.” Sources included original studies, clinical guidelines, consensus statements, and high-quality reviews, with an emphasis on relevance to older adults and applicability to non-dental clinicians. Dental caries in older adults commonly presents as root and recurrent decay and isstrongly associated with xerostomia, polypharmacy, chronic disease, functional impairment, cognitive decline, and limited access to dental care. The literature supports fluoride-based prevention, management of salivary dysfunction, dietary counseling, minimally invasive therapies such as silver diamine fluoride, and interprofessional care models. Non-dental providers play a critical role in screening, risk assessment, preventive counseling, symptom triage, and referral. Integrating oral health into routine medical care is an essential and achievable strategy to reduce morbidity and improve health outcomes in older adults.

## 1. Introduction

Older adults are among the fastest-growing demographic groups in the United States and worldwide [1]. By 2030, 1 in 6 people globally will be over 60 years old, and in the United States, 1 in every 5 residents, or more than 70 million Americans, will be 65 years or older [2]. As the population ages, the burden of chronic disease increases. Dental caries or cavities are among the most common chronic diseases affecting older adults. Nearly all adults aged 65 years or older have had a cavity [3], and about 12.5% have at least one untreated cavity [4].

Managing dental caries is important because of its significant impact on health. Caries can cause pain and tooth loss, which can affect speech, self-esteem, and nutrition and adversely affect overall health and quality of life [5,6,7]. Untreated caries and resulting infections can also exacerbate systemic conditions like diabetes and cardiovascular disease [7]. Although physiologic changes associated with aging increase the risk of dental decay, caries is not an inevitable consequence of aging. Prevention and early intervention can reduce caries morbidity [8].

Despite its impact on health, a high percentage of older adults with unmet dental needs do not visit a dentist annually [9,10]. Many older adults who do not visit a dentist will see a non-dental provider [11], presenting opportunities to improve oral health. However, oral health issues are frequently overlooked by primary care providers [12,13]. One reason may be gaps in knowledge and a lack of awareness among health professionals of the importance of dental decay.

This narrative review seeks to address these gaps by summarizing the current literature related to dental caries and older adults. Specifically, this review aims to enhance awareness of the impact of oral health on older adults and address deficiencies in non-dental providers’ knowledge. These aims should help non-dental providers to develop strategies and practices to improve their patients’ oral health.

## 2. Methods

A narrative literature review provides a broad synthesis of the literature using a non-systematic approach to summarize current understanding on a topic [14]. Unlike systematic reviews, which address narrowly pre-specified questions [15,16], narrative reviews are suited for exploring expansive topics. Accordingly, narrative reviews serve as a valuable tool for concisely distilling complex information, making them useful for examining comprehensive subjects such as dental caries in the older adults [17,18].

To develop the structure of this review, two authors (MSL and MH) independently conducted a literature search, using the MeSH terms “dental caries,” OR “root caries,” AND “aged,” to formulate an initial draft outline. After developing their respective outlines, they compared findings and collaborated to produce a final version.

The finalized outline guided non-systematic searches on PubMed and Google Scholar from January 2000 through June 2026 for English-language publications with an emphasis on more recent publications. The search combined the MeSH terms “dental caries”, “root caries, “aged”, “aged 80 and over” and “primary health care” with free-text keywords. Keywords included epidemiology, pathophysiology, risk factors, clinical presentation, screening and diagnosis in non-dental settings, prevention, and treatment. The authors used Boolean operators (AND/OR) to refine and expand the search as appropriate.

The authors assessed the retrieved articles for relevance to older adults and applicability to non-dental clinicians and manually reviewed the bibliographies of selected studies to identify additional relevant sources. The authors gave preference to original research, clinical practice guidelines, consensus statements, and high-quality review articles. When applicable, the authors searched authoritative public health and governmental sources, such as the Centers for Disease Control and Prevention and the National Institutes of Health, for epidemiological and statistical data and major organizations such as the American Academy of Family Physicians and the American Dental Association for policy and guideline recommendations.

Sources reviewed included clinical practice guidelines, consensus documents, original research articles, review articles, government-sponsored websites, and additional sources such as case studies, professional organization statements, and peer-reviewed commentaries reflecting on the role of non-dental providers in maintaining oral health. The review excluded trade publications, non-authoritative websites, and publications lacking empirical or policy-relevant content. Excluding references for methodology and census data, the authors used 85 sources, including 39 review articles, 24 original research studies, 10 consensus or organizational statements, 4 organizational or governmental websites, 6 commentaries, and 2 book chapters.

To enhance credibility and methodological rigor, the authors applied the Scale for the Assessment of Narrative Review Articles (SANRA), a critical appraisal tool for narrative reviews. SANRA assesses six components: (1) justification of the review’s relevance, (2) clear articulation of its objectives, (3) thorough description of the literature search strategy, (4) targeted and appropriate referencing, (5) sound scientific reasoning, and (6) comprehensive presentation of relevant data and conclusions [19]. Two authors (MH and MSL) independently assessed the final manuscript using SANRA criteria and reconciled discrepancies through discussion to ensure the review met SANRA criteria and maintained a high quality. Supplementary Table S1 outlines adherence to the SANRA criteria.

## 3. Pathophysiology

### 3.1. Definition and Disease Process

Dental caries is a biofilm-mediated, dynamic disease characterized by the net loss of minerals from dental hard tissues [20] and influenced by behavioral, psychological, and environmental factors [21]. It is not merely a structural defect or “hole,” but rather a chronic biochemical process occurring at the interface between the tooth surface and the oral biofilm. The essential pathophysiologic event is an imbalance in which demineralization exceeds remineralization over time [22,23]. This imbalance results from interactions among oral microorganisms, fermentable carbohydrates, host factors such as saliva, and the structural properties of tooth tissues [24].

In older adults, the distribution of disease shifts from primarily coronal caries to root surface and recurrent caries [25,26]. This transition is related to gingival recession, which exposes dentin and cementum following cumulative periodontal disease, mechanical abrasion, or age-related changes in the periodontium. These root tissues are less mineralized than enamel, making them more vulnerable to acid dissolution [27]. As a result, lesions in older adults may develop more rapidly, progress with less obvious early symptoms, and occur in sites that are more difficult for patients and clinicians to recognize. This risk may be further amplified by difficulties in maintaining proper hygiene [28].

### 3.2. Demineralization and Remineralization

The progression of caries is determined by the balance between mineral loss and mineral replacement [29]. Cariogenic bacteria within dental plaque, particularly acidogenic and aciduric organisms such as *Streptococcus mutans* and *Lactobacillus* species, metabolize fermentable carbohydrates to produce organic acids [30]. These acids lower the pH at the tooth–biofilm interface and promote dissolution of hydroxyapatite crystals [20]. Enamel begins to demineralize at a pH of approximately 5.5, whereas root surfaces are susceptible at a higher pH, ranging from 6.2 to 6.7. This higher critical pH helps explain why exposed roots in older adults are especially prone to rapid decay [16].

Saliva is central to the maintenance of oral mineral homeostasis. It buffers acids, dilutes carbohydrates, provides calcium and phosphate ions, and facilitates remineralization of early lesions [31,32]. Saliva also contributes to oral clearance, lubrication, and antimicrobial defense. However, in older adults, salivary protection can be compromised. Hyposalivation and xerostomia are common consequences of polypharmacy, particularly with anticholinergic medications, antidepressants, antipsychotics, antihypertensives, and other drugs frequently prescribed in later life [33,34]. When salivary flow is reduced, the oral environment becomes more acidic, less buffered, and less capable of repairing early mineral loss, thereby accelerating lesion formation and progression [20].

### 3.3. Natural History and Clinical Progression

Caries develops on a continuum. Early lesions begin as subsurface demineralization that may be reversible if the biofilm is disrupted and mineral balance restored. Without intervention, the lesion enlarges, cavitation develops, and bacterial invasion extends into dentin and eventually the pulp. Progressive disease may culminate in irreversible pulpitis, pulpal necrosis, periapical infection, abscess formation, and tooth fracture or loss [35].

In older adults, cumulative restorations, exposed root surfaces, impaired dexterity, poor dental hygiene practices, and delayed access to dental care amplify caries progression [36]. Recurrent caries around existing restorations is common and may reflect longstanding plaque retention, marginal breakdown, or persistent exposure to cariogenic conditions [37]. Because early lesions are often asymptomatic, the disease may go unnoticed until significant structural destruction or infection has already occurred.

### 3.4. Clinical and Systemic Consequences

Untreated caries has important local, functional, systemic, and psychosocial consequences [20,38]. Locally, the disease can cause pain, sensitivity, infection, halitosis, and progressive structural destruction of teeth. Functionally, caries and tooth loss impair chewing efficiency, often leading patients to select softer, more processed, carbohydrate-rich foods. This dietary adaptation may increase caries risk while contributing to poor nutrition [39,40].

Systemically, poor oral health can interact bidirectionally with chronic disease [41,42,43]. Oral infection and inflammation may complicate glycemic control in patients with diabetes, while aspiration of pathogenic oral microorganisms may increase pneumonia risk in frail or institutionalized older adults [44]. In addition, chronic oral pain and functional decline can reduce independence and undermine overall well-being. The impact of dental caries in later life is therefore not confined to teeth; it extends to systemic health, nutritional status, and quality of life [45].

### 3.5. Risk Amplifiers in Older Adults

The burden of caries in older adults reflects the convergence of biological, behavioral, and social vulnerability. Common contributing factors include root exposure, chronic disease, polypharmacy, dry mouth, high-frequency carbohydrate intake, reduced dexterity, cognitive impairment, limited caregiver support, and barriers to dental access [46]. These overlapping factors create a sustained imbalance in favor of demineralization, making dental caries one of the most common and clinically significant oral diseases of later life [43].

## 4. Prevention

Prevention of dental caries in older adults requires a comprehensive and individualized approach that reflects the realities of aging, including physiologic change, multimorbidity, medication burden, and functional limitation. Successful prevention is rarely achieved through a single intervention; rather, it depends on combining daily oral hygiene, fluoride exposure, dietary modification, salivary support, and practical behavior-change strategies [47,48].

Effective plaque control remains the foundation of prevention. Patients should be encouraged to brush twice daily with fluoride toothpaste and to perform regular interdental cleaning when feasible [49]. For individuals at elevated risk, particularly those with root exposure, xerostomia, or prior caries experience, high-concentration fluoride toothpaste may be especially beneficial [50,51,52]. Older adults with arthritis, neurologic disease, frailty, or cognitive impairment may have difficulty performing routine oral hygiene independently [52]. In these situations, adaptive aids such as power toothbrushes, modified handles, floss holders, and caregiver-assisted brushing can substantially improve plaque removal and the consistency of care [52].

Dietary counseling is another key preventive strategy. Many older adults shift toward softer foods that are easier to chew or prepare, but these choices may be highly processed and rich in fermentable carbohydrates. Clinicians should emphasize not only the amount of sugar consumed, but also the frequency of exposure, because repeated between-meal carbohydrate intake prolongs the acidic challenge to tooth surfaces. Limiting frequent snacking, reducing sugary beverages, and encouraging less cariogenic alternatives can meaningfully reduce risk [53,54].

Management of xerostomia is critical because reduced salivary flow undermines the oral environment’s natural defenses. Medication review should be a routine component of prevention, with attention to cumulative anticholinergic burden and other drugs associated with dry mouth [55]. When clinically appropriate, modifying, substituting, or deprescribing contributing medications may improve symptoms. Additional measures include frequent hydration, avoidance of alcohol-containing rinses, and the use of salivary stimulants such as sugar-free gum or xylitol-containing lozenges [55]. For more severe salivary dysfunction, saliva substitutes and sialogogues may offer symptomatic relief.

Fluoride remains the cornerstone of evidence-based caries prevention in older adults because it inhibits demineralization and supports remineralization [56]. High-fluoride toothpaste, professionally applied fluoride varnish, and fluoride mouth rinses are particularly useful in high-risk individuals [57]. Adjunctive agents such as silver diamine fluoride and calcium phosphate-based remineralizing products may be considered in selected patients, especially for nonoperative management of early or root lesions [58]. Taken together, prevention in older adults should be proactive, multimodal, and tailored to individual risk, with the explicit goal of preserving function, reducing treatment burden, and preventing avoidable pain and infection. Table 1 summarizes the core preventive priorities for non-dental providers.

## 5. Treatment

### 5.1. A Shift Toward Medical Management

Historically, caries treatment was often viewed as synonymous with operative dentistry. In contemporary geriatric oral health care, however, the framework has broadened to include “medical management of caries,” an approach focused on disease control, lesion arrest, risk reduction, and preservation of tooth structure whenever possible [59,60]. This paradigm is especially relevant in older adults, for whom frailty, comorbidity, transportation barriers, and treatment tolerance may complicate traditional restorative care.

### 5.2. Nonoperative Management

Nonoperative therapy seeks to arrest disease progression without surgical removal of tooth structure. This strategy is particularly valuable for frail, homebound, cognitively impaired, or medically complex patients, and for those with limited access to dental treatment. Silver diamine fluoride (SDF) is one of the most important advances in this area. As a topical antimicrobial and remineralizing agent, SDF can effectively arrest many root caries lesions while avoiding the pain, cost, and procedural burden of conventional treatment [61,62,63]. Although black staining of arrested lesions limits its aesthetic appeal, this tradeoff is often acceptable in posterior teeth or in patients for whom stabilization is the highest priority [64].

Prescription-strength fluoride toothpaste is another important tool for older adults at high risk, especially those with xerostomia or exposed root surfaces [20,65,66]. In addition, management of dry mouth should be part of caries treatment, not merely symptom relief, because restoration of salivary function directly addresses the disease environment. For vulnerable older adults, successful treatment may therefore consist of arresting active disease, reducing risk factors, and preventing progression rather than immediately pursuing invasive restorative procedures.

### 5.3. Operative Management

Operative intervention is indicated when carious lesions are cavitated, trap food, compromise function, or cause symptoms that cannot be managed conservatively. In older adults, minimally invasive approaches are often preferred [67,68]. Atraumatic restorative treatment involves the removal of soft infected tissue with hand instruments, followed by restoration with a material such as glass ionomer cement [69,70]. This method is less traumatic than conventional drilling, may be more feasible in community or bedside settings, and offers the additional benefit of fluoride release.

More extensive restorative approaches, including composite restorations, crowns, or endodontic therapy, may be appropriate in selected patients with adequate functional reserve, oral hygiene capacity, and treatment tolerance. However, treatment planning in older adults should always be individualized and balance longevity of restoration with patient goals, cognitive status, medical stability, anticipated follow-up, and caregiver support. The most technically elaborate procedure is not always the most appropriate intervention in geriatric care [71].

### 5.4. Urgency and Triage

For non-dental providers, one important clinical task is determining the urgency of referrals. An asymptomatic dark spot or cavitated lesion warrants routine dental referral, although high-risk patients may benefit from interim preventive measures such as high-fluoride toothpaste while awaiting evaluation. Sensitivity to sweets, cold, or heat suggests a more active process and should prompt referral within one to two weeks. Persistent or spontaneous dental pain raises concern for pulpal involvement and merits urgent dental assessment within 24 to 48 h. Facial swelling, fever, trismus, dysphagia, or difficulty breathing signal spreading infection and require emergency evaluation because of the risk of deep space infection or sepsis [43].

Table 2 presents a practical triage framework for non-dental providers.

## 6. Role of the Non-Dental Provider

Non-dental providers, including primary care clinicians, geriatricians, nurses, pharmacists, and other allied health professionals, play a pivotal role in the prevention, identification, and initial management of dental caries in older adults [72]. As older individuals access medical care more frequently than dental services, routine clinical encounters represent critical and often underutilized opportunities to address oral health. Incorporating oral health into standard medical care can help reduce unmet dental needs [73], identify disease at earlier stages, and mitigate downstream complications.

### 6.1. Screening and Risk Identification

Non-dental health providers can integrate brief oral assessments into routine medical visits without incurring a considerable time burden. Visual inspection of the oral cavity should include evaluation for visible decay, plaque accumulation, gingival inflammation, recession exposing root surfaces, broken teeth, and ill-fitting prostheses. In addition, targeted symptom inquiry, such as pain, thermal sensitivity, difficulty chewing, halitosis, or dry mouth, can provide valuable diagnostic insight and prompt further evaluation [74].

Risk assessment is equally important and should be systematic. Key factors include polypharmacy (particularly medications with anticholinergic effects), diabetes and other chronic conditions, reduced salivary flow, prior caries experience, functional limitations affecting oral hygiene, cognitive impairment, and social determinants such as limited access to care or caregiver support. Identifying individuals at elevated risk enables clinicians to implement targeted preventive strategies and prioritize timely referral, thereby preventing progression to more advanced disease [73].

### 6.2. Preventive Interventions

Non-dental providers are well-positioned to initiate evidence-based preventive interventions. High-fluoride toothpaste (5000 ppm sodium fluoride) is effective in reducing the incidence and progression of root caries in high-risk older adults [51,75] and can be prescribed in medical settings. Additional preventive strategies include dietary counseling to reduce frequent sugar exposure, reinforcement of oral hygiene practices, and management of xerostomia [76].

Medication review is particularly important, as many commonly prescribed drugs contribute to reduced salivary flow [76]. Where feasible, minimizing anticholinergic burden or adjusting medication regimens may improve oral conditions [77]. Supportive measures such as hydration, salivary stimulants (e.g., xylitol-containing products), and saliva substitutes can also be recommended.

In select settings, particularly in long-term care facilities, community clinics, or underserved areas, non-dental providers may also play a role in the application of topical agents such as fluoride varnish or silver diamine fluoride. These interventions can be highly effective in preventing or arresting caries in patients who face barriers to accessing traditional dental care [78].

### 6.3. Referral and Care Coordination

Timely referral to dental professionals is essential for definitive diagnosis and management. Non-dental providers should be able to distinguish between routine and urgent presentations. Progressive pain, suspected infection, facial swelling, or systemic symptoms warrant urgent or emergent referral, while asymptomatic lesions can typically be managed with routine dental follow-up [73].

Establishing clear referral pathways with dental providers, particularly those experienced in geriatric, community-based, or special care dentistry, can improve access and continuity of care. This is especially important for medically complex or vulnerable populations, including long-term care residents, homebound individuals, and patients with cognitive impairment, for whom oral health needs are frequently unmet [79].

### 6.4. Interprofessional Collaboration

An interprofessional approach is essential to improving oral health outcomes in older adults. Collaboration among medical providers, dentists, pharmacists, nurses, and caregivers allows for comprehensive management of shared risk factors, including xerostomia, dietary habits, and chronic disease [80]. Pharmacists can assist with medication optimization, nurses and caregivers can support daily oral hygiene, and dental professionals can provide definitive treatment.

Integrating oral health into routine medical workflows, such as incorporating oral assessments into annual wellness visits or chronic disease management encounters, has been increasingly recommended as a strategy to address persistent gaps in care [81]. By embedding oral health into general healthcare delivery, non-dental providers can play a transformative role in reducing the burden of dental caries and improving overall health in aging populations.

### 6.5. Special Populations

Certain subgroups of older adults face substantial risk for dental caries and often require tailored management strategies. Residents of long-term care facilities are among the most vulnerable [82]. Many depend on staff for daily oral hygiene, yet oral care may be inconsistent because of staff turnover, inadequate training, time constraints, or competing care priorities. As a result, plaque accumulation, untreated decay, and oral discomfort may persist unnoticed for prolonged periods.

Older adults with dementia or other forms of cognitive impairment also represent a high-risk group [83]. These individuals may lose the ability to perform oral hygiene independently, forget instructions, have difficulty identifying or reporting pain, or resist care because of fear, confusion, or sensory intolerance. Consequently, the disease may progress silently until advanced infection, eating difficulty, or behavioral changes emerge. In these patients, caregiver involvement is not optional but central to effective prevention and early detection.

In palliative care and advanced illness, the goals of oral health management change. Rather than prioritizing comprehensive restorative treatment, the emphasis shifts to comfort, symptom relief, infection control, and preservation of dignity. Xerostomia, mucosal discomfort, candidiasis, and painful teeth may all be important targets for intervention. Minimally invasive and nonoperative approaches are often the most appropriate in this context because they align with patient-centered goals and reduce procedural burden.

Denture wearers also warrant special attention. Although dentures replace missing teeth, they do not eliminate oral disease risk. Poor denture hygiene can promote biofilm accumulation and contribute to denture stomatitis, candidiasis, halitosis, and inflammation of surrounding tissues [84]. In patients with partial dentures or remaining natural teeth, retained biofilm may also increase the risk of caries on adjacent tooth surfaces. Across all special populations, successful management depends on simplified routines, caregiver training, realistic prevention plans, and consistent attention to oral hygiene as part of overall care. Table 3 summarizes practical roles for non-dental providers across prevention, triage, and care coordination.

## 7. Limitations

Narrative reviews provide a useful overview of the literature for a complex topic. However, since they do not use a systematic search strategy, there is a risk of selection bias and omission of relevant studies. Also, narrative reviews rely on the authors’ interpretations rather than a formal appraisal of the evidence. To mitigate these limitations, the authors applied SANRA narrative review criteria to enhance the review’s rigor and credibility.

## 8. Literature Gaps

Despite organizational efforts and recommendations to incorporate oral health into primary care [85,86,87], little evidence documents the effectiveness of this approach for older adults. Most of the literature focuses on younger adults and pediatric settings [88], and although evidence supports collaborative benefits [89], more research is needed to document its effectiveness in older adults [90]. Seamless collaboration between oral health providers and other clinicians requires efficient bidirectional communication, and more research exploring best practices for EHR communication are needed to break down silos [91]. One study found that dentists’ use of mobile electronic communications facilitated improved oral health behaviors and oral health knowledge among older adults [92], and exploring this technology with non-dental providers or integrating it to enhance patient-provider communication in medical settings represents a research opportunity.

## 9. Conclusions

Dental caries in older adults is a common, progressive, and consequential disease that remains prevalent despite being potentially preventable. As more adults retain their natural dentition into advanced age, the burden of root and recurrent caries continues to increase. Xerostomia, polypharmacy, chronic disease, functional limitation, cognitive decline, and barriers to dental access all contribute to this growing problem.

The significance of caries in later life lies not only in tooth destruction, but also in its wider effects on pain, nutrition, systemic health, social participation, and quality of life. For these reasons, oral health is an integral component of comprehensive geriatric care rather than a separate or secondary concern. Non-dental providers are positioned to improve outcomes because they encounter older adults regularly and have opportunities to identify risks long before advanced dental complications arise.

Through brief oral assessments, thoughtful medication review, dietary and xerostomia counseling, fluoride-based prevention, and timely referral, clinicians outside dentistry can make a meaningful contribution to reducing disease burden. Interprofessional collaboration is especially important for complex, frail, and access-limited patients. Integrating oral health into routine medical practice is both practical and necessary, and it represents a key step toward healthier aging, better function, and improved overall well-being for older adults.

## Figures and Tables

**Table 1:** Key Preventive Strategies for Dental Caries in Older Adults.

Preventive Domain	Clinical Focus	Examples for Practice
Daily plaque control	Reduce biofilm accumulation and promote consistent hygiene	Twice-daily brushing with fluoride toothpaste; daily interdental cleaning when feasible; caregiver-assisted hygiene
Adaptive oral hygiene	Support patients with functional or cognitive limitations	Power toothbrushes, modified handles, floss holders, simplified routines
Dietary counseling	Lower frequency of acid exposure and fermentable carbohydrate intake	Reduce between-meal sugar, limit sugary/acidic beverages, and encourage less cariogenic alternatives
Xerostomia management	Restore oral protection by improving salivary function and symptom control.	Medication review, hydration, xylitol products, saliva substitutes, sialogogues when appropriate
Fluoride therapy	Enhance remineralization and reduce progression of early lesions	High-fluoride toothpaste, fluoride varnish, fluoride mouthrinse
Adjunctive nonoperative therapy	Stabilize lesions in selected high-risk patients	Silver diamine fluoride; calcium-phosphate remineralizing agents

**Table 2:** Triage and Referral for Suspected Dental Caries in Older Adults.

Clinical Presentation	Concern	Recommended Action
Asymptomatic dark spot, visible cavitation, or food trapping without pain	Nonurgent active lesion	Routine dental referral; consider preventive measures such as high-fluoride toothpaste while awaiting care
Sensitivity to cold, heat, or sweets	Active dental lesion or early pulpal irritation	Prompt dental referral, ideally within 1–2 weeks
Persistent pain or spontaneous throbbing pain	Possible pulp involvement or advancing infection	Urgent dental evaluation within 24–48 h; analgesics as appropriate
Facial swelling, fever, dysphagia, dyspnea, trismus	Spreading odontogenic infection	Immediate referral to the emergency department or oral and maxillofacial surgery

**Table 3:** Practical Role of the Non-Dental Provider in Dental Caries Care.

Issue	Non-Dental Provider Role
Caries recognition	Understand the impact of untreated caries on pain, nutrition, systemic health, and quality of life.
Prevention	Recommend routine dental care; counsel about brushing, interdental cleaning, and diet; consider high-fluoride toothpaste or mouthrinse for high-risk patients.
Xerostomia	Review medications; consider alternatives or reduction in xerogenic burden when feasible; recommend hydration and xylitol-containing products.
Nonoperative management	In selected settings, consider fluoride varnish or training in silver diamine fluoride use for patients with limited access to dental services.
Referral	Refer asymptomatic lesions routinely; expedite referral for sensitivity or progressing symptoms; recognize emergencies requiring immediate evaluation.
Special populations	Support caregiver education; recommend adaptive devices such as modified toothbrush handles, power brushes, or water flossers when appropriate.

## Data Availability

No new data were created or analyzed in this study. Data sharing is not applicable to this article.

## Supplementary Materials

The following supporting information can be downloaded at: https://www.mdpi.com/article/10.3390/ijerph23080965/s1, Table S1: Adherence to the Scale for the Assessment of Narrative Review Articles (SANRA) Criteria.
