# Supplementary material for: Dental Caries in Older Adults: A Narrative Review for Non-Dental Providers
Source: Int J Environ Res Public Health. 2026 Jul 26;23(8):965. doi: 10.3390/ijerph23080965 (PMC13512720; doi:10.3390/ijerph23080965)
Supplement: Supplementary file 1 [file ijerph-23-00965-s001.zip › ijerph-4347182-supplementary.pdf]

Table S1. Adherence to the Scale for the Assessment of Narrative Review Articles (SANRA) Criteria.

Criterion	Approach	Rationale
Justification of the article's importance for the readership	A narrative review is well suited to synthesizing a broad clinical topic for a multidisciplinary readership.	Dental caries represents a significant health burden, particularly among older adults. Non-dental clinicians can help identify risks, reinforce preventive measures, and facilitate access to appropriate dental care.
Statement of concrete aims	Two authors independently conducted literature searches using the MeSH terms "dental caries," "root caries," and "aged" and jointly developed a structured outline that defined the review's objectives and scope.	The review aims to address knowledge gaps, increase awareness of the clinical importance of oral health, and provide practical recommendations for non-dental clinicians caring for older adults.
Description of the literature search	Guided by the structured outline, the authors conducted a targeted, non-systematic search of PubMed and Google Scholar for English-language publications from January 2000 through June 2026, with an emphasis on more recent publications. MeSH terms were used in PubMed, with corresponding free-text keywords used in Google Scholar. The search terms included "dental caries," "root caries," "aged," "aged 80 and over," and "primary health care." Additional search concepts included epidemiology, pathophysiology, risk factors, clinical presentation, screening and diagnosis in non-dental settings, prevention, and treatment, with a focus on older adults.	Reporting the databases, publication period, language restrictions, search concepts, and population of interest provides transparency while remaining appropriate for a narrative review.
Referencing	Priority was given to original research, clinical guidelines, consensus documents, and high-quality review articles, supplemented by selected governmental and professional organization sources, including the CDC, NIH, American Academy of Family Physicians (AAFP), and ADA. The reference lists of retrieved articles were also reviewed to identify additional relevant sources.	Sources were selected on the basis of methodological credibility, clinical relevance to non-dental clinicians, and alignment with the review's objectives and structure.
Scientific reasoning	The authors synthesized findings across source types according to the structured outline, emphasizing the consistency, clinical relevance, and applicability of the evidence to non-dental practice.	This approach links the cited evidence to the review's factual statements, interpretations, and clinical recommendations rather than relying on unsupported opinion.
Appropriate presentation of relevant data	Relevant epidemiologic, clinical, preventive, and treatment findings were selected and presented to support the narrative synthesis, with particular attention to evidence applicable to older adults.	Concrete and clinically meaningful findings were used, when available, to support key statements, conclusions, and recommendations.

Note: Two authors independently assessed the completed narrative review using the SANRA instrument and reconciled discrepancies through discussion.
